# Supplementary material for: A Population Coupling Model Identifies Reduced Propagation from V1 to Higher Visual Areas During Locomotion
Source: bioRxiv. 2026 Feb 6:2026.02.04.703681. Preprint. [Version 1] doi: 10.64898/2026.02.04.703681 (PMC12889648; doi:10.64898/2026.02.04.703681)
Supplement: Supplement 1 [file NIHPP2026.02.04.703681v1-supplement-1.pdf]

bioRxiv preprint doi: https://doi.org/10.64898/2026.02.04.703681; this version posted February 6, 2026. The copyright holder for this preprint (which was not certified by peer review) is the author/funder, who has granted bioRxiv a license to display the preprint in perpetuity. It is made available under aCC-BY 4.0 International license.

Supplementary materials

Lack of refractory effects in linear self-history filters of population spike trains

In this section, we show that the refractory effect, a transient period after a spike during which the neuron's likelihood to fire again is reduced, can become less pronounced at the population level after pooling individual spike trains. This discrepancy can have significant consequences when modeling with GLMs. Specifically, a population-level model may exhibit instability even if individual neurons are stable.

Consider a population consisting of N_A GLM neurons. Each neuron in the population only receives self-history effects from itself. For simplicity, we will assume that all neurons share an identical self-history filter h and a constant baseline β_0.

For each neuron, its behavior can be described as:

$$\log \lambda_i = h * Y_i + \beta_0, \tag{5}$$

where λ_i is the firing rate of the i-th neuron, and Y_i represents its binary spike train.

Let $Y = \sum_{i=1}^{N_A} Y_i$ denote the population spike train. The population firing rate is given by

$$\lambda = \sum_{i}^{N_A} \lambda_i. \tag{6}$$

We can derive the firing rate of the population spike train as:

$$\log \lambda = \log \left(\sum_{i=1}^{N_A} \lambda_i \right) \tag{7}$$

$$= \beta_0 + \log \left(\sum_{i}^{N_A} \exp(h * Y_i) \right). \tag{8}$$

Due to the convexity of exponential function, we have the following inequation

$$\sum_{i}^{N_A} \exp(h * Y_i) \geq N_A \cdot \exp(h * Y/N_A). \tag{9}$$

Thus,

$$\log \lambda \geq \log(N_A) + \beta_0 + h * Y/N_A \tag{10}$$

$$= \tilde{\beta_0} + Y * \tilde{h}, \tag{11}$$

where $\tilde{\beta_0} = \log(N_A) + \beta_0$ and $\tilde{h} = h/N_A$ represent the linearly scaled baseline and post-spike filter.

bioRxiv preprint doi: https://doi.org/10.64898/2026.02.04.703681; this version posted February 6, 2026. The copyright holder for this preprint (which was not certified by peer review) is the author/funder, who has granted bioRxiv a license to display the preprint in perpetuity. It is made available under aCC-BY 4.0 International license.

This inequality implies that the true log firing rate of the population, $\log \lambda$, is greater than the prediction provided by the linearly scaled parameters $\tilde{\beta}_0$ and $\tilde{h}$. Therefore, if we fit a population GLM to the data to obtain estimated parameters $\hat{\beta}_0$ and $\hat{h}$, these estimates must compensate for this underestimation. Consequently, compared to the theoretically scaled parameters $\tilde{\beta}_0$ and $\tilde{h}$, at least one of the following assertions regarding the fitted estimates must hold true:

- The estimated baseline is higher than the linearly scaled prediction ($\hat{\beta}_0 > \tilde{\beta}_0$).
- The estimated population self-history filter $\hat{h}$, which is typically negative during the refractory period, is weaker (less negative) than the scaled individual filter $\tilde{h}$.

In our simulation, we consistently observed that the fitted population self-history filter, denoted by $\hat{h}$, is substantially weaker than $\tilde{h}$ when the post-spike filters of individual neurons emulate biologically authentic refractory effects (Figure S1A). Consequently, the overall refractory effects in the population-level GLMs, given by $\hat{h} * \tilde{Y}$, are much weaker than $\tilde{h} * \tilde{Y} \approx h * Y_i$, which represents the refractory effects in a single neuron. This suggests that there tends to be a lack of refractory effects if we just use a linear self-history filter to capture refractory effects in population spike trains.

Next, we highlight a scenario where an individual neuron remains stable, but the model tailored to the population spike trains exhibits instability due to this lack of refractory effects. In this model, we introduce excitatory coupling effects within the population of N_A GLM neurons. Similarly, we assume that every neuron receives coupling filters from all other neurons in the group and all coupling filters are consistent, represented by c. Figure S1B shows the generated spike trains, where the ground truth single-neuron GLM never explodes. However, if we fit population-level GLM with a linear self-history filter and inhomogeneous baseline to the population spike train, it becomes unstable, as the fitted self-history filter does not reflect strong refractory effects and the self-history filter is dominated by excitatory coupling effects (Figure S1D). The fact that linear self-history filters at population level fail to capture enough refractory effects explains why the nonlinear correction term in our pop-GLM is very significant and prevents explosion.

Correction for multiple comparisons

Given that we are examining 30 directed coupling pairs of areas, it is necessary to adjust for multiple comparisons. The 30 significance tests are not independent, making the Bonferroni correction potentially conservative [62]. To get an exact permutation-based adjustment, we used the following procedure.

In our results, the corrected p-values using Bonferroni and this exact procedure were close, likely due to the near independence of the 30 tests [62].

bioRxiv preprint doi: https://doi.org/10.64898/2026.02.04.703681; this version posted February 6, 2026. The copyright holder for this preprint (which was not certified by peer review) is the author/funder, who has granted bioRxiv a license to display the preprint in perpetuity. It is made available under aCC-BY 4.0 International license.

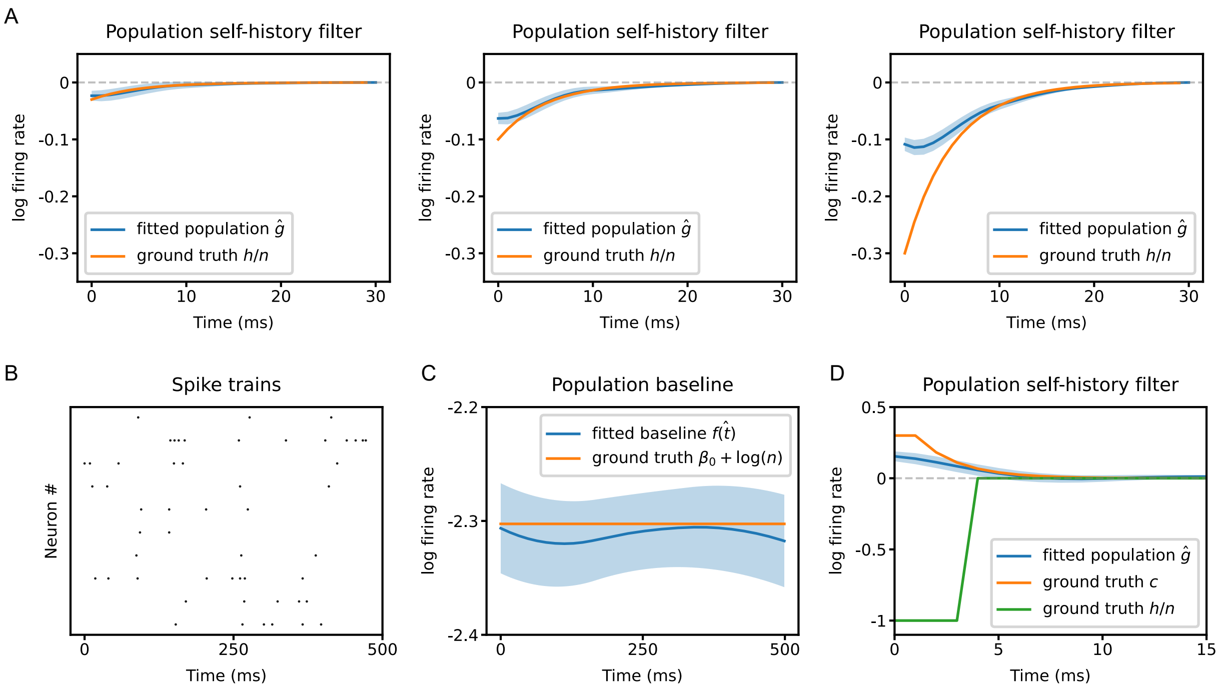

Figure S1: Explanation of why population spike trains lack refractory effects when using linear self-history filters and require additional nonlinear correction. **A**, Simulate ten independent GLM neurons with a post-spike filter h and a constant baseline, then fit a GLM with a self-history filter $\hat{g}$ on population spike trains. From left to right: increasing refractory effects. $\min(h) = -0.3$, -1, and -3, respectively. The fitted population self-history filter $\hat{g}$ is much higher than h/N_A for biologically realistic refractory effects, where a spike would decrease the log firing rate by 2 or 3. **B, C, D**, an example case where an individual neuron does not explode, but a population-level GLM fitted on the population spike train would explode. First, generate spike trains from a population of ten GLM neurons (**B**). There are both post-spike filters for each individual neuron, and a coupling filter from any neuron to any other neuron. Then fit a GLM on the population spike train with two components: inhomogeneous baseline (**C**) and self-history effects (**D**). The fitted population self-history filter is entirely above zero, and we don't observe any refractory effects. Such strong positive feedback from post-spikes leads the model to explode.

bioRxiv preprint doi: https://doi.org/10.64898/2026.02.04.703681; this version posted February 6, 2026. The copyright holder for this preprint (which was not certified by peer review) is the author/funder, who has granted bioRxiv a license to display the preprint in perpetuity. It is made available under aCC-BY 4.0 International license.

Algorithm 2: Testing Procedure

Input: Test statistics for 30 cases under 82600 permutations $T_{i,n}$ $(i = 1, 2, ..., 30;\ n = 1, 2, ..., 82600)$; observed test statistics for 30 cases T_i^{obs}

Output: Corrected p-values $p'_{(1)}$, $p'_{(2)}$, ... for the most significant cases

1. Calculate the nominal p-value for each case. Identify the smallest nominal p-value, denoted by $p_{(1)}$
2. For each permutation $n = 1, 2, \ldots, 82600$, compute the nominal p-values for the set of test statistics $T_{:,n}$. Record the frequency at which the smallest p-value is less than $p_{(1)}$, which is denoted as the corrected p-value $p'_{(1)}$.
3. Exclude the case associated with $p_{(1)}$, leaving 29 cases. Repeat step 2, using $p_{(2)}$ in place of $p_{(1)}$, to determine $p'_{(2)}$.
4. Continue as in step 3 for the i-th smallest p-value $p_{(i)}$, until the corrected p-value $p'_{(i)}$ exceeds 0.05.

Model Selection for Self-History Effects

This section provides a detailed exploration of the model selection process, specifically focusing on addressing the explosion issue (Figure 1C). These explorations are summarized in Table S2, where various model configurations are compared based on their Bayesian Information Criterion (BIC) improvements and stability.

Initially, we identified that both cross-area coupling and self-history effects are necessary and significantly improve the model's fit. However, as indicated in the main text, adding self-history effects led to instability. To address this, we explored various modifications to the model.

One approach was the inclusion of additional terms to the log firing rate. We introduced a new covariate, $\Lambda(t) = \frac{1}{\tau}\sum_{k=1}^{n} \exp(-(t - t_k^*)/\tau)$, capturing the empirical spike density over a short history window. Variations of this term were tested, including Λ itself, its quadratic form Λ^2, and a nonparametric term $f_{\text{damp}}(\Lambda)$. Among these, the nonparametric term $f_{\text{damp}}(\Lambda)$ stood out for its robust ability to prevent explosion and significantly improve the fit, as detailed in the main text and shown in Table S2.

Another pathway we explored was adding the dependence between the empirical spike density $\Lambda(t)$ and self-history filters, rather than directly adding a term to the log firing rate. Specifically, we modified the fixed self-history filter $\mathbf{g}$ to a varying version $\widetilde{\mathbf{g}(\Lambda)} = \mathbf{g_0} + \Lambda \cdot \mathbf{g_1}$, which depends on Λ, allowing for more flexible adaptation to the spike train's history. In this way, the total self-history effects becomes:

bioRxiv preprint doi: https://doi.org/10.64898/2026.02.04.703681; this version posted February 6, 2026. The copyright holder for this preprint (which was not certified by peer review) is the author/funder, who has granted bioRxiv a license to display the preprint in perpetuity. It is made available under aCC-BY 4.0 International license.

Model name and equation	BIC improvement comparing to baseline
Baseline without considering speed: $\log \lambda_t = f_t$	0
Linear term of instantaneous speed: $\log \lambda_t = f_t + \beta \cdot s_t$	75.09
Binary term of instantaneous binary state: $\log \lambda_t = f_t + \beta \cdot I(s_t > v_{th})$	6.93
Two-way coupling of instantaneous speed: $\log \lambda_t = f_t + \mathbf{k}_{speed} \cdot \mathbf{s}_{(t-l):(t+l)}$	109.12
Time-dependent term of instantaneous speed: $\log \lambda_t = f_t + \beta_t \cdot s_t$	555.69
Time-dependent term of instantaneous binary state: $\log \lambda_t = f_t + \beta_t \cdot I(s_t > v_{th})$	914.43
**Time-dependent term of trial-wise binary state:** $\boldsymbol{\log \lambda_t = f_t + \beta_t \cdot I(\bar{s} > v_{th})}$	**1454.99**

Table S1: Model selection with regard to speed. In our model selection process concerning speed, we fit models incorporating the effects of speed in various ways and compare their Bayesian Information Criterion (BIC). The model with the trial-wise binary state provides the best BIC, which is highlighted in bold. According to this model, a trial is classified as a running trial if its average speed exceeds 1 cm/s. Conversely, trials with an average speed below this threshold are classified as stationary. In our notation, λ_t denotes the firing rate at the t-th time bin, and f_t is the time-varying baseline. The term s_t represents the recorded running speed at time t, and β is the fitted coefficient. For defining the binary state, I is the indicator function, and v_{th} is the speed threshold used to classify states as either stationary or running. The vector $\mathbf{k}_{speed}$ serves as the filter associated with the recent past and future running speeds. β_t is the non-stationary coefficient related to instantaneous speed or state. In all models listed in the table, we have also considered the optimal lead-lag time between the firing rate λ_t and running speed s_t, as well as the best smoothing parameter for running speed s_t, determined through cross-validation. v_{th} is another hyperparameter, which was optimized using cross-validation. In practice, a v_{th} value of 1 provided the best fit, resulting in an equal distribution of running and stationary trials.

bioRxiv preprint doi: https://doi.org/10.64898/2026.02.04.703681; this version posted February 6, 2026. The copyright holder for this preprint (which was not certified by peer review) is the author/funder, who has granted bioRxiv a license to display the preprint in perpetuity. It is made available under aCC-BY 4.0 International license.

Model components				BIC improvement	Stability
Baseline	Coupling	Self-history	Correction term		
✗				0	Yes
✗	✗			4362.17	No
✗	✗	✗		5941.72	No
✗	✗	✗ (with first-order dependence)		8183.60	Yes
✗	✗	✗ (with second-order dependence)		7682.57	No
✗	✗	✗	✗ (linear)	6383.48	No
✗	✗	✗	✗ (quadratic)	8094.84	Yes
**✗**	**✗**	**✗**	**✗ (nonparametric)**	**8929.0**	**Yes**

Table S2: Comparison of models that consider coupling/history effects. I decided to use the model with coupling, history, and Λ^2 based on AIC and stability. The outcome of the GLM is probe C (i.e. V1) pooled spike trains. In this table, "coupling" means the coupling effects (coupling filter convolved with the spike trains of another area) from all other five areas; history effect means the post-spike filter convolved with the spike trains of itself; 1st and 2nd order correction on history/coupling mean I allow the post-spike filter/ coupling filter to change when different Λ; Λ^2 just means adding Λ^2 to the right-hand-side of the equation as a predictor. Besides the predictors in the table, the GLMs also include the time-warped inhomogeneous baseline and the trial-wise gain constant. I fit running and stationary models separately to account for speed's effects, then add the AIC of the two models together. Stability is estimated by doing simulations, both fragile (sometimes explode) and divergent (always explode) are labeled as "No" in the last column of stability. Details and equations for these models are in Supplementary.

$$
\begin{aligned}
h(t|H_t) &= \mathbf{Y} * \tilde{\mathbf{g}} && (12)\\
&= \mathbf{Y} * (\mathbf{g_0} + \Lambda \cdot \mathbf{g_1}) && (13)\\
&= \mathbf{Y} * \mathbf{g_0} + \Lambda \cdot \mathbf{Y} * \mathbf{g_1} && (14)\\
&= \sum_{k=1}^{n} g_0(t - t_k^*) + \Lambda \cdot \sum_{k=1}^{n} g_1(t - t_k^*). && (15)
\end{aligned}
$$

We also considered second-order interactions by substituting $\mathbf{g}$ with a more complex formulation of $\tilde{\mathbf{g}}(\Lambda) = \mathbf{g_0} + \Lambda \cdot \mathbf{g_1} + \Lambda^2 \cdot \mathbf{g_2}$. Although incorporating first-order dependency improved the fit and stabilized the model, it did not surpass the enhancements brought by the nonparametric term added to the log firing rate. Therefore, for its robustness, adaptability, and simplicity, we ultimately chose the nonparametric term $f_{\text{damp}}(\Lambda)$ as our final model.

bioRxiv preprint doi: https://doi.org/10.64898/2026.02.04.703681; this version posted February 6, 2026. The copyright holder for this preprint (which was not certified by peer review) is the author/funder, who has granted bioRxiv a license to display the preprint in perpetuity. It is made available under aCC-BY 4.0 International license.

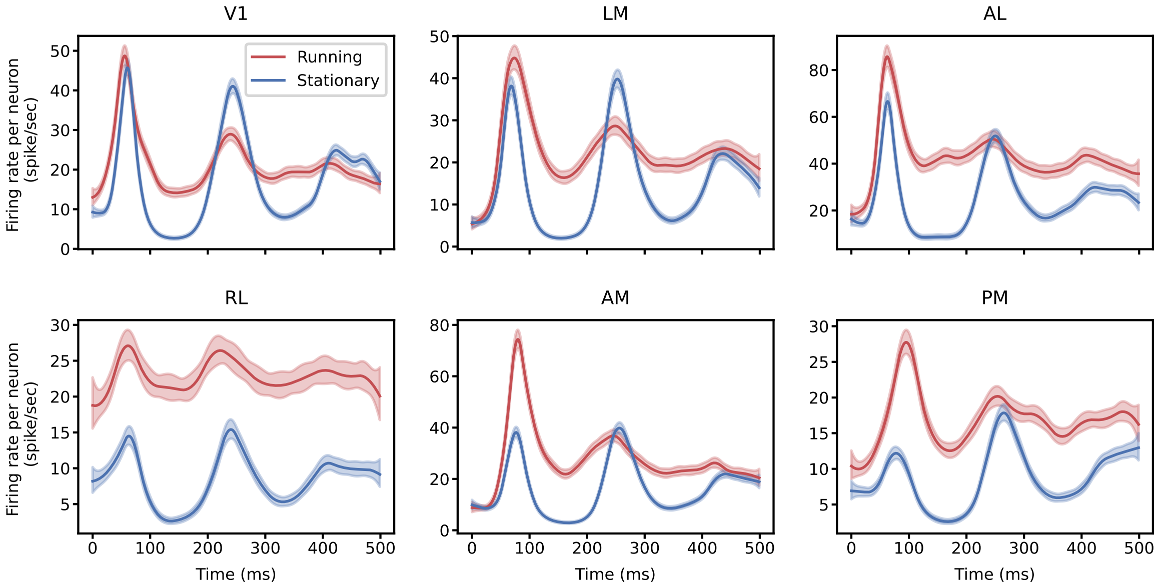

Figure S2: Same as Figure 5, but using all available neurons in each area. Mean firing rates of six populations in six areas, under running and stationary conditions.

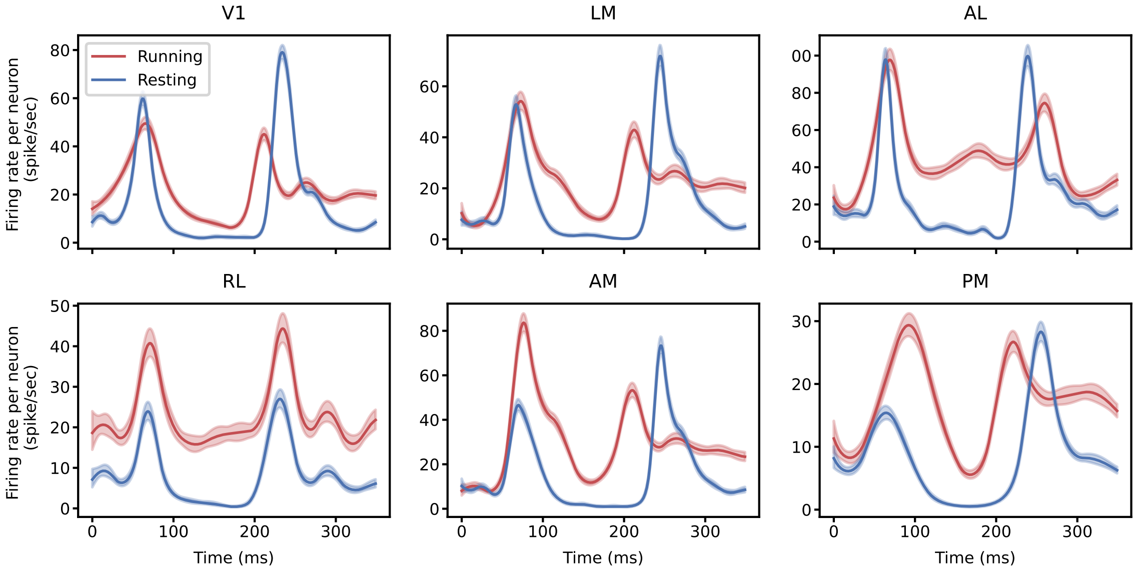

Figure S3: Firing rate templates $f_j(t)$ from models with only time-warped inhomogeneous baseline ($\log \lambda_{j,m}(t) = f_j(\phi_{j,m}^{-1}(t))$). The firing rate template is thinner than the mean firing rate in Fig5.

bioRxiv preprint doi: https://doi.org/10.64898/2026.02.04.703681; this version posted February 6, 2026. The copyright holder for this preprint (which was not certified by peer review) is the author/funder, who has granted bioRxiv a license to display the preprint in perpetuity. It is made available under aCC-BY 4.0 International license.

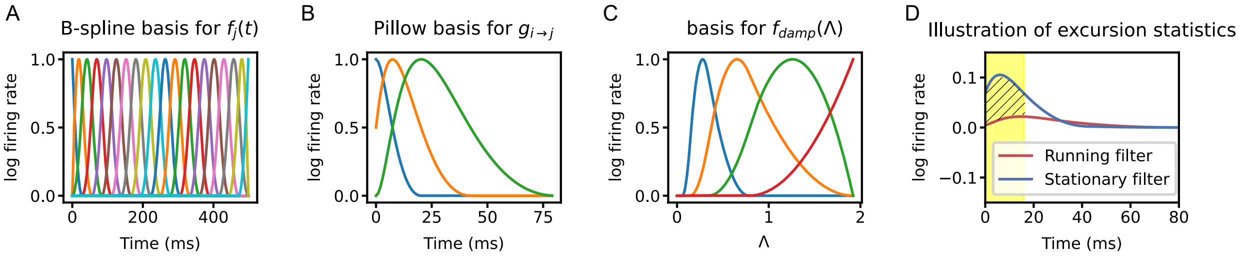

Figure S4: **A**, Basis functions for the inhomogeneous baseline template $f_j(t)$ consist of 30 B-spline bases. **B**, Basis functions for the cross-area coupling filter $g_{i\to j}$ consist of three Pillow bases. **C**, Basis functions for the population refractory function $f_{\text{damp}}\Lambda$ consist of four basis functions. They do not start from zero to avoid non-identification issues and are not evenly spaced because there are much fewer data points when Λ is large. **D**, Illustration of excursion statistics. The yellow area shows the region where the difference between the two curves is greater than half the maximum difference, or the region of interest. The excursion statistic is defined as the area of the shaded region.

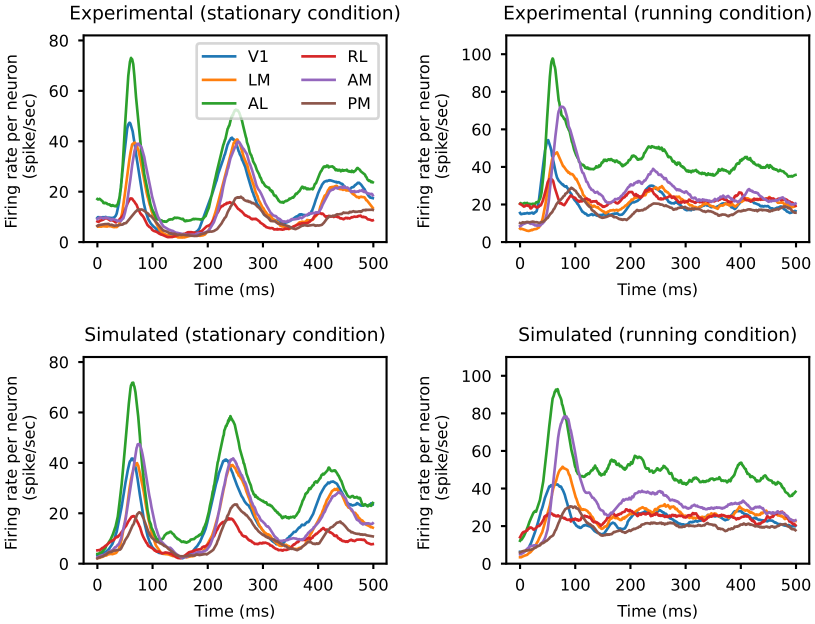

Figure S5: Simulated PSTH looks similar to experimental PSTH. **A&B**, Experimental PSTH at stationary and running conditions. **C&D**, PSTH of simulated spike trains generated by stationary models and running models.

bioRxiv preprint doi: https://doi.org/10.64898/2026.02.04.703681; this version posted February 6, 2026. The copyright holder for this preprint (which was not certified by peer review) is the author/funder, who has granted bioRxiv a license to display the preprint in perpetuity. It is made available under aCC-BY 4.0 International license.

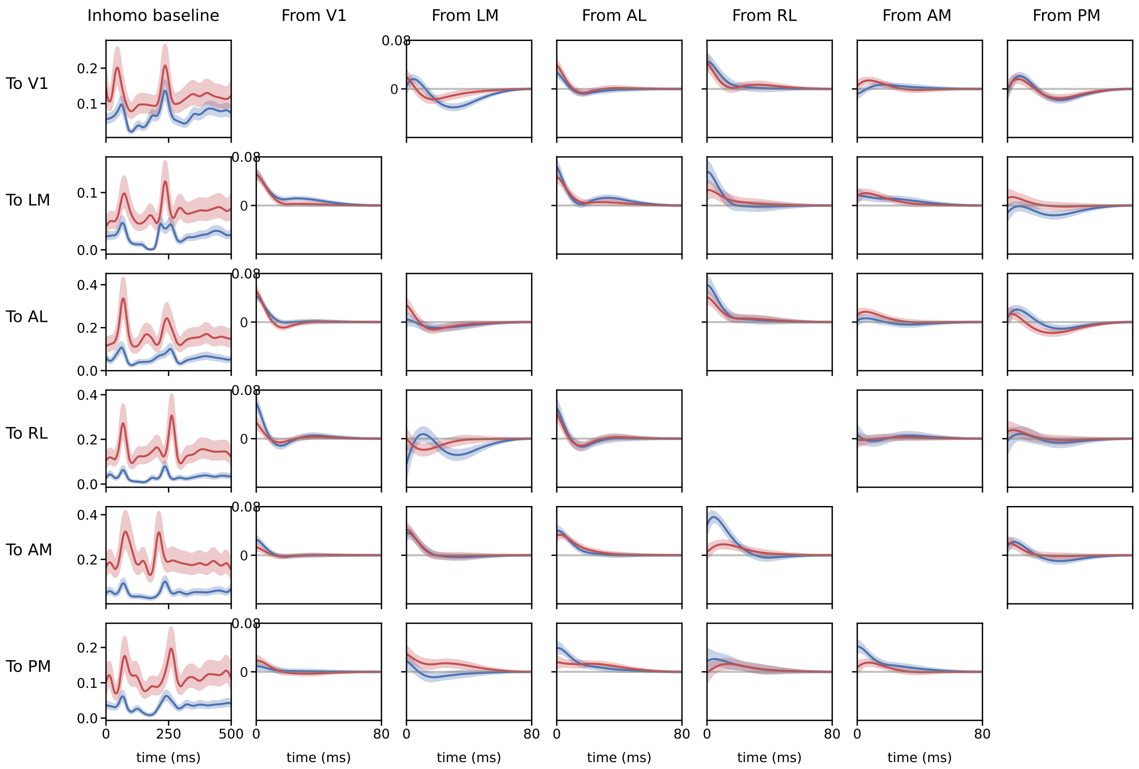

Figure S6: Same as Figure 6, but using all available neurons in each area.

bioRxiv preprint doi: https://doi.org/10.64898/2026.02.04.703681; this version posted February 6, 2026. The copyright holder for this preprint (which was not certified by peer review) is the author/funder, who has granted bioRxiv a license to display the preprint in perpetuity. It is made available under aCC-BY 4.0 International license.

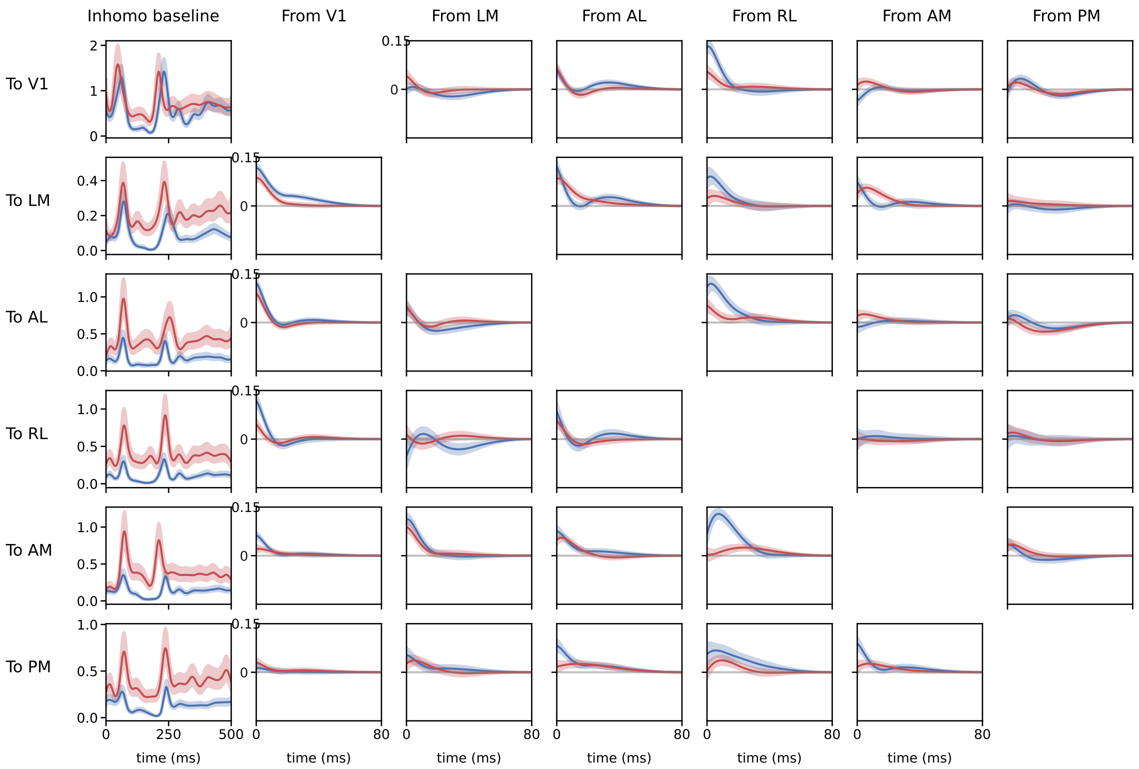

Figure S7: Same as Figure 6, but without including the nonlinear correction term f_{damp} in the model. Instead, we estimate each neuron's self-history filter h_l with single-neuron GLMs using data from -0.5s to 0s, with 0 indicating the onset of the stimulus. Then we calculate the exact total single-neuron history effects $\log \sum_{l=1}^{N} \exp(h_l * Y_l)$ for all neurons in the population during 0-0.5s, and use it as a fixed offset when fitting pop-GLMs. The filters here are very similar to the filters in Figure 6, indicating that the nonlinear history effects at the population level are an adequate approximation to the total history-dependent effects of individual neurons.

bioRxiv preprint doi: https://doi.org/10.64898/2026.02.04.703681; this version posted February 6, 2026. The copyright holder for this preprint (which was not certified by peer review) is the author/funder, who has granted bioRxiv a license to display the preprint in perpetuity. It is made available under aCC-BY 4.0 International license.

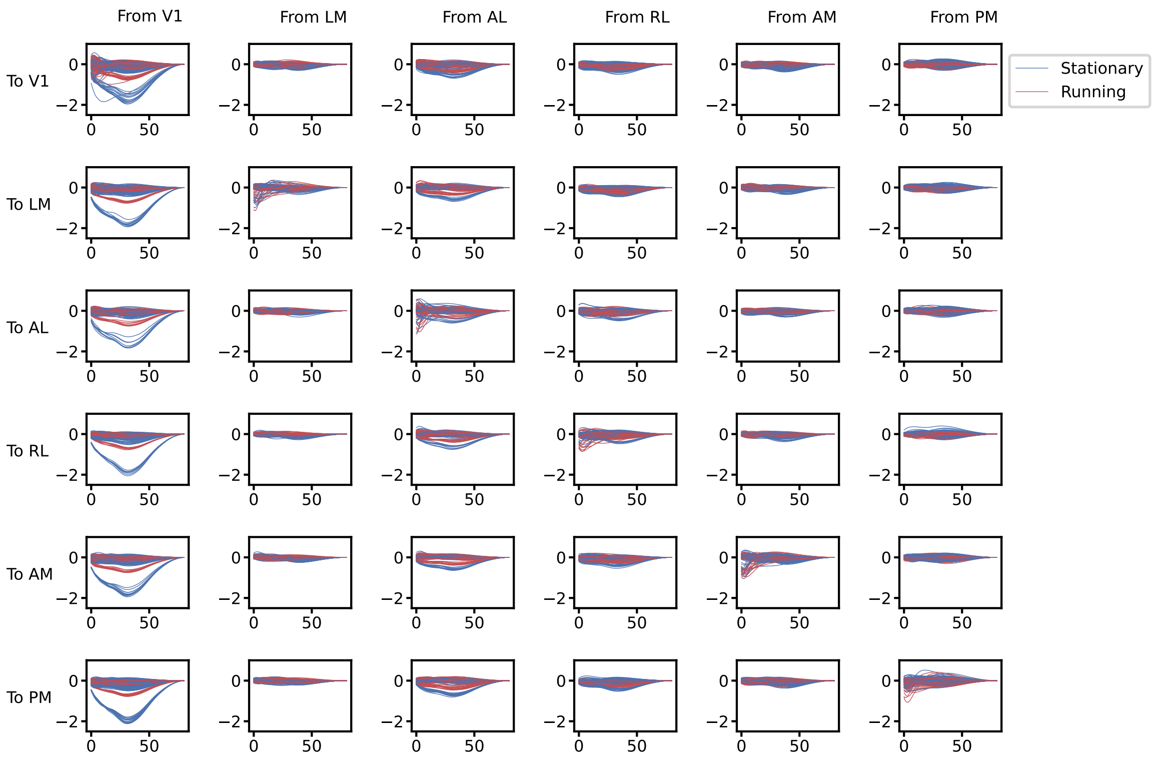

Figure S8: Fitted coupling filters using Huk et al.'s method to Allen Institute's dataset. The coupling filters from V1 to all other areas are the most significant ones and are inhibitory.

bioRxiv preprint doi: https://doi.org/10.64898/2026.02.04.703681; this version posted February 6, 2026. The copyright holder for this preprint (which was not certified by peer review) is the author/funder, who has granted bioRxiv a license to display the preprint in perpetuity. It is made available under aCC-BY 4.0 International license.

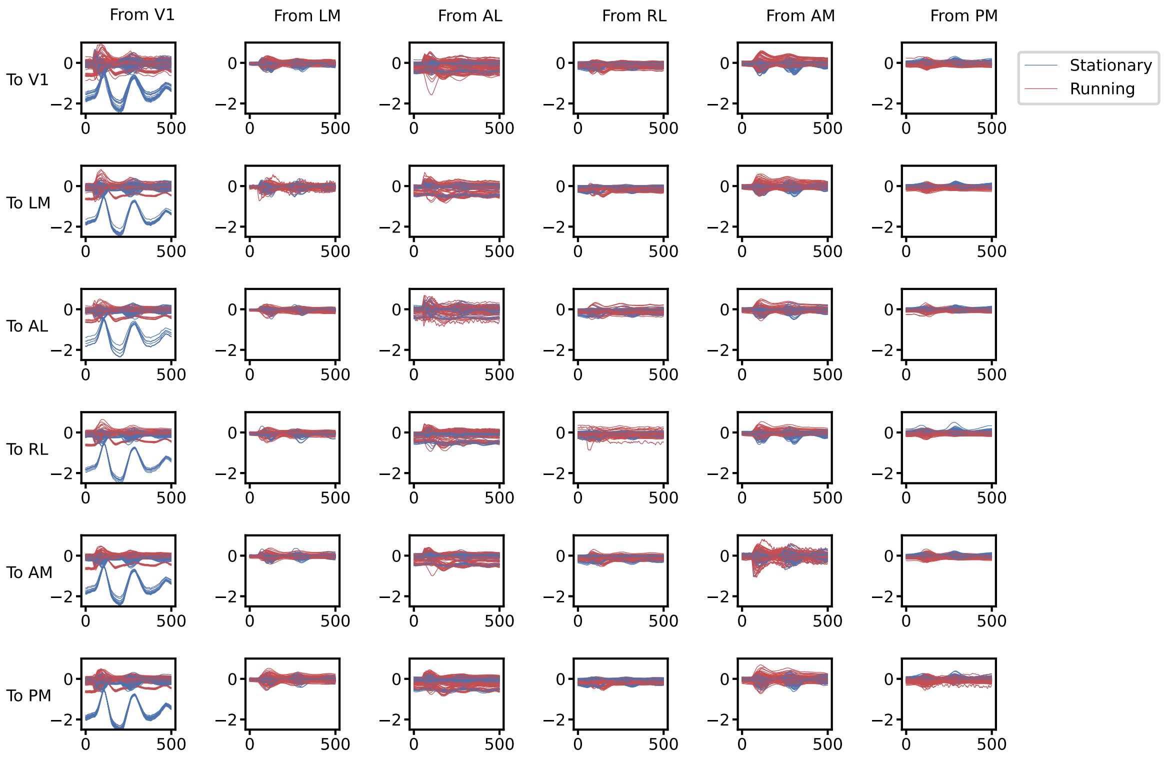

Figure S9: Fitted coupling effects' outputs using Huk et al.'s method. The single-neuron-level model suggests that V1's inhibition to other areas dominates the firing rates. The two firing rate peaks result from V1's low activity tens of milliseconds ago. These fitted results do not seem reasonable.

bioRxiv preprint doi: https://doi.org/10.64898/2026.02.04.703681; this version posted February 6, 2026. The copyright holder for this preprint (which was not certified by peer review) is the author/funder, who has granted bioRxiv a license to display the preprint in perpetuity. It is made available under aCC-BY 4.0 International license.

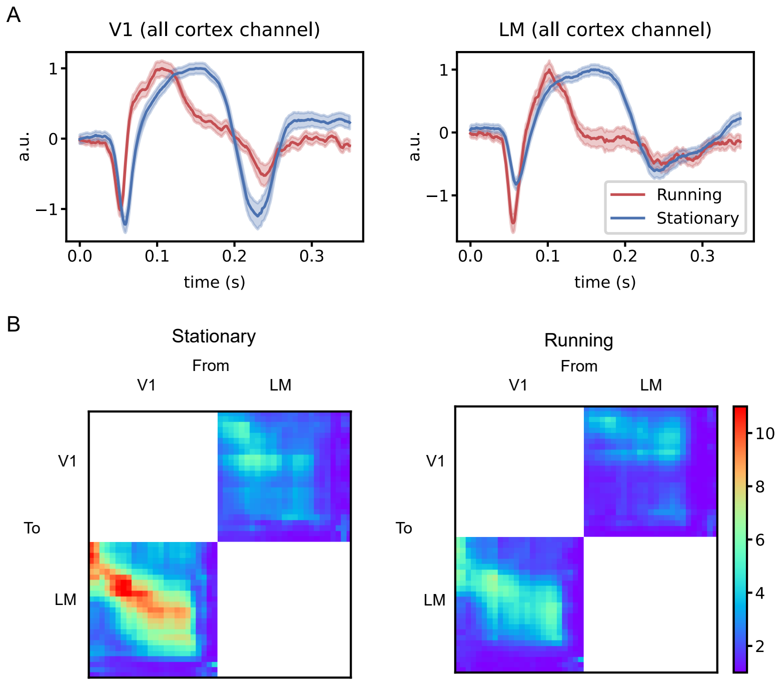

Figure S10: **A**, Trial-averaged Local Field Potential (LFP) post-stimulus onset under both stationary and running conditions. The LFP is averaged across multiple channels within an area. Left: V1. Right: LM. Under stationary conditions, two peaks are observed, whereas the second peak is less prominent when running, aligning with the spiking intensity observed in Figure 5. **B**, Trial-averaged Granger causality test statistics from V1 channels to LM channels. Left: stationary conditions. Right: running conditions. The Granger causality test statistics reveal a stronger connection from V1 to LM than from LM to V1, and these statistics notably diminish during running. The order of the Granger causality test is five, determined by AIC, and we ensured the significance in the tests is not due to nonstationarity by detrending and visualization.

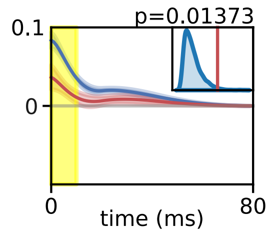

Figure S11: Fitted coupling filter from V1 to LM for the second validation session. We directly applied the analysis from the first session without altering any hyperparameters. The significant connection change from V1 to LM, observed in the first mouse, is also substantiated in the second mouse.

bioRxiv preprint doi: https://doi.org/10.64898/2026.02.04.703681; this version posted February 6, 2026. The copyright holder for this preprint (which was not certified by peer review) is the author/funder, who has granted bioRxiv a license to display the preprint in perpetuity. It is made available under aCC-BY 4.0 International license.

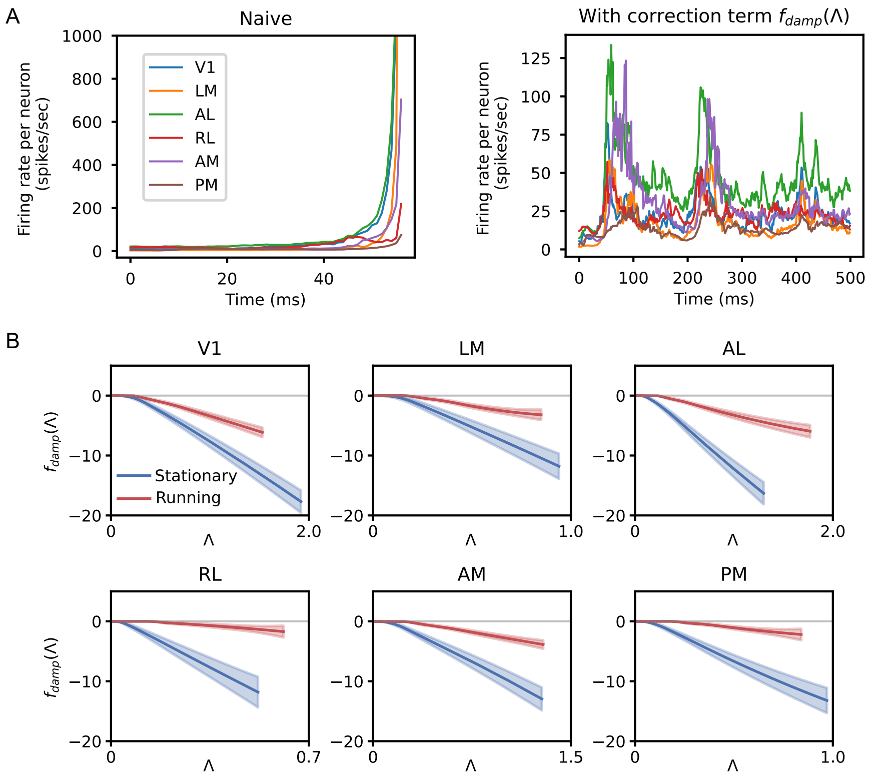

Figure S12: **A**, Simulated firing rates of six areas using models with and without the nonlinear correction term f_{damp}. **B**, Fitted f_{damp} in the six areas under stationary and running conditions.

bioRxiv preprint doi: https://doi.org/10.64898/2026.02.04.703681; this version posted February 6, 2026. The copyright holder for this preprint (which was not certified by peer review) is the author/funder, who has granted bioRxiv a license to display the preprint in perpetuity. It is made available under aCC-BY 4.0 International license.

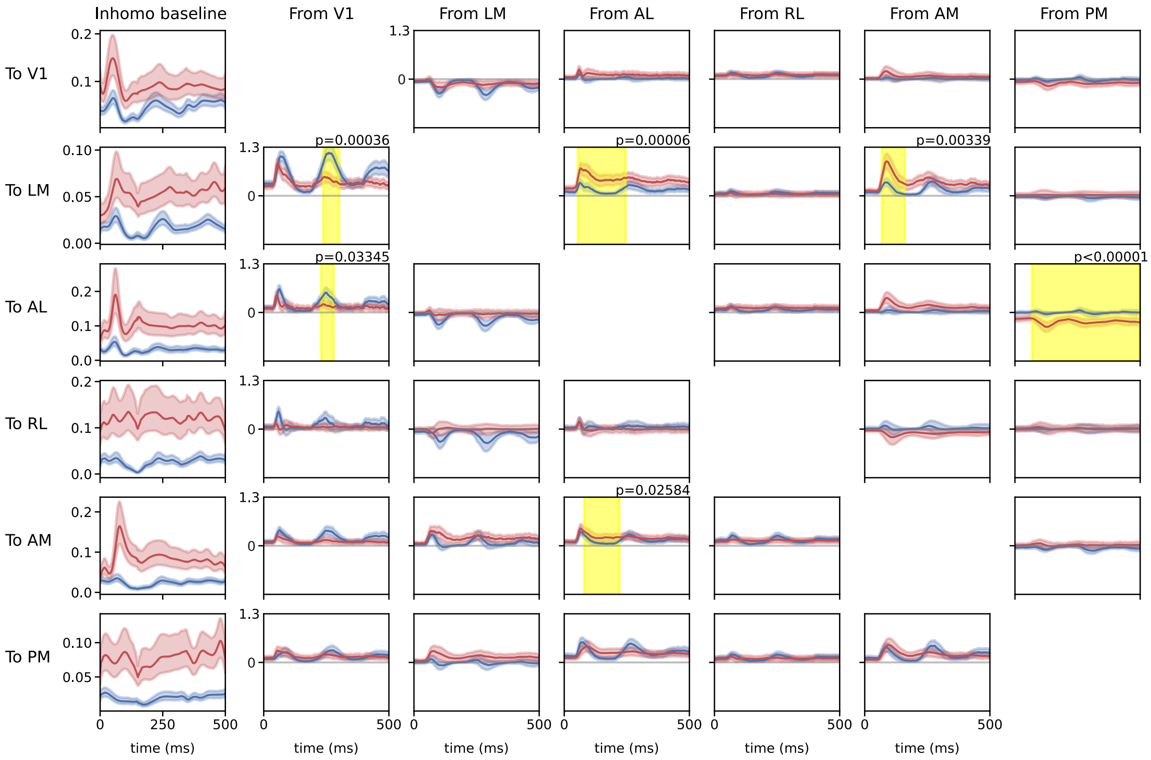

Figure S13: Fitted GLM outputs of each component, averaged across trials (95% CI). Only coupling effects and time-warped inhomogeneous baselines are shown. Red: running. Blue: stationary. Excursion tests were performed on the two curves in each panel, but the null distribution is not shown here. Yellow areas indicate the selected regions found by excursion tests with p-values below 0.05. For example, the first row shows the response variable as V1 population spike trains, with coupling effects from all six areas and an inhomogeneous baseline (interpreted as input from the thalamus and other unobserved sources).
